# A Very Uncommon Case of Transudative Chylothorax: A Case Report and Literature Review

**DOI:** 10.7759/cureus.38320

**Published:** 2023-04-30

**Authors:** Fahed Owda, Shatha Mallah, Mohammed Ayyad, Maram Albandak, Shahed Yousef, Alaa Hmeedan, Mahmoud Odeh, Adam M Reid, Wadi Sleibi, Jehad Azar

**Affiliations:** 1 Internal Medicine, An-Najah National University, Nablus, PSE; 2 Internal Medicine, Al-Quds University, Jerusalem, PSE; 3 School of Medicine, Al-Quds University, Jerusalem, PSE; 4 Research, University of California Los Angeles, California, USA; 5 Respiratory Institute, Cleveland Clinic, Cleveland, USA

**Keywords:** pleurodesis, thoracentesis, cirrhosis, liver dysfunction, chylothorax

## Abstract

The presence of chyle in the pleural cavity is referred to as chylothorax. Exudative chylothorax is usually related to damage or obstruction of the lymphatic vasculature with subsequent leakage into the pleural space. In contrast, transudative chylothorax is related to increased hydrostatic pressure caused by elevated intra-abdominal pressure, which leads to the translocation of chylous fluid into the pleural space. Cirrhosis is the most common cause of transudative chylothorax, commonly presenting with ascites and portal hypertension. To the best of our knowledge, isolated transudative chylothorax as a consequence of cirrhosis is exceptionally rare and has been scarcely reported in the literature. We herein report a female patient in her fifties who presented to our hospital with isolated unilateral transudative hepatic chylothorax, with no clinical evidence of cirrhosis or any stigmata of portal hypertension at the time of presentation.

## Introduction

Chylothorax is defined as the accumulation of chyle in the pleural space and confirmed by thoracocentesis showing a triglycerides concentration of more than 110 mg/dL or positive chylomicrons in the pleural fluid [1,2]. Chylothorax formation has an extensive list of traumatic and non-traumatic etiologies. Approximately 50% of chylothorax cases are due to traumatic etiologies and can be further classified as surgical causes, with esophagectomy and corrective operations for congenital heart disease being the most common, and non-surgical causes such as thoracic radiation, and blunt trauma [3,4]. On the other hand, some non-traumatic causes of chylothorax include malignancies, which account for 17%-46% of all cases, with lymphoproliferative malignancies being the most common. Lymphatic anomalies, sarcoidosis, infections such as tuberculosis, and transdiaphragmatic movement of chylous ascites in the context of liver cirrhosis constitute the majority of the remaining causes [3,5,6].

Although the predominant cases of chylothorax were described as exudative pleural effusions, in rare instances, transudative chylothoraces have been reported in alcoholic and non-alcoholic liver cirrhosis, congestive heart failure, nephrotic syndrome, amyloidosis, superior vena cava syndrome, sclerosing mesenteritis, lymphangioleiomyomatosis, and tuberculosis [7-9]. The most likely explanation for the development of chylothorax in cirrhotic patients is closely linked to the transdiaphragmatic movement of chyle into the pleural cavity through diaphragmatic pores, created by pleuro-peritoneal blebs, which tend to occur more commonly in the right hemidiaphragm [10].

The prevalence and incidence of isolated hepatic hydro-chylothorax in the absence of clinical and radiological evidence of ascites are scarce and pose a distinctive diagnostic challenge [11]. In this paper, we present a unique case of isolated unilateral rapidly re-accumulating high-output transudative chylothorax in a patient with a history of chronic alcohol abuse, without clinical evidence related to cirrhosis or portal hypertension.

## Case presentation

A female patient in her fifties, with a past medical history significant for alcohol abuse, depression, and tobacco use disorder, presented to our department with a two-week history of progressive dyspnea worse with exertion. She denied having fever, hemoptysis, weight loss, or a history of similar episodes. On admission, her vital signs were stable, but she was noted to be in mild respiratory distress. She had no ascites, peripheral edema, jugular venous distention, jaundice, or any other signs suggestive of heart failure or liver disease. Chest examination revealed decreased breath sounds and dullness to percussion over the entire right hemithorax. Her blood work, including liver enzymes and synthetic liver function tests, was unremarkable.

**Figure 1:**
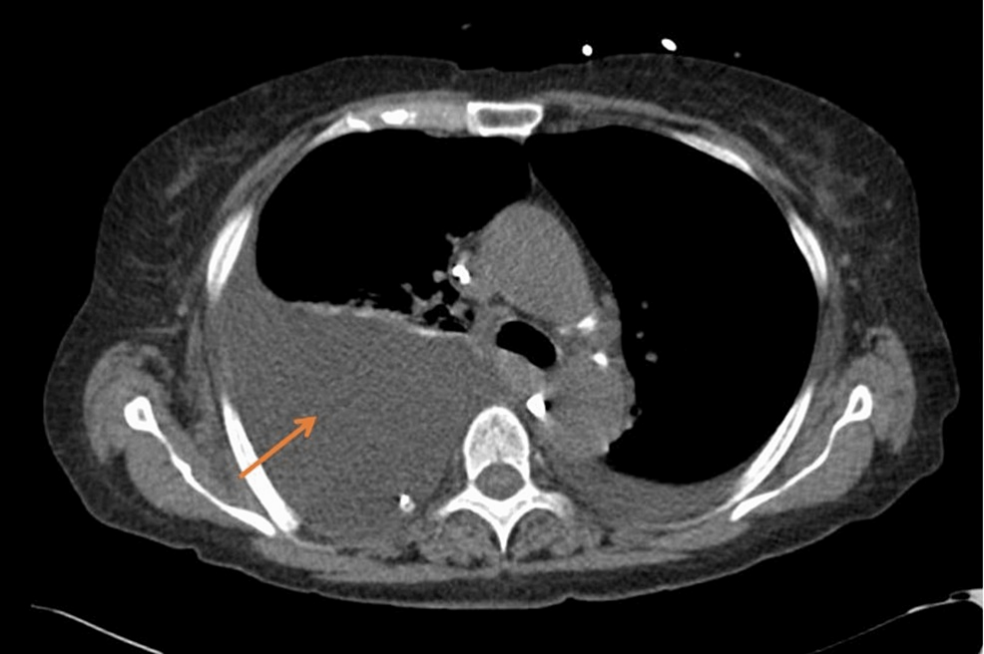
CT scan of the chest on admission CT scan of the chest showing a large right-sided pleural effusion with compressive atelectasis (orange arrow) and a small left-sided pleural effusion

Chest X-ray and computed tomography (CT) scan confirmed the presence of a large right-sided pleural effusion (Figure 1). Abdominal ultrasound (U/S) showed cholelithiasis and gallbladder sludge without secondary signs of acute cholecystitis. No biliary dilation, splenomegaly, or ascitic fluid was noted. Thoracocentesis was done and returned two liters of milky-colored fluid. Pleural fluid analysis results were consistent with a transudative type chylothorax (Table 1).

**Table 1:** Pleural fluid analysis findings on thoracocentesis LDH: lactate dehydrogenase; TG: triglycerides

Pleural fluid analysis		Normal values
Pleural/serum LDH ratio	0.4	Transudative ≤0.6
Pleural/serum protein ratio	0.3	Transudative ≤0.5
TG (mg/dL)	165 mg/dL	<110 mg/dL
Cholesterol (mg/dL)	< 25 mg/dL	<45 mg/dL
Amylase (U/L)	24 U/L	<160 U/L
Eosinophils (%)	14	<10%
Lymphocytes (%)	59	10-40%

The patient reported marked improvement following pleural drainage. Additionally, a bedside ultrasound confirmed the resolution of her effusion. However, a few hours later, she began suffering from dyspnea and right-sided chest pain. A chest X-ray showed recurrent accumulation of fluid in the right pleural space. Subsequently, she underwent a repeated therapeutic thoracentesis, followed by chest tube placement. She was clinically deteriorating and had high output chest tube drainage, resulting in hypovolemic shock and severe electrolyte imbalance. A central venous catheter was placed and aggressive intravenous resuscitation was initiated. The patient was eventually placed on total parenteral nutrition (TPN) due to high chest tube output leading to hypovolemic shock with electrolytes imbalance.

Extensive investigations revealed no cardiac, renal, or hepatic causes of transudative effusion and no evidence of nephritic or nephrotic syndromes. Echocardiography showed normal biventricular function with a left ventricular ejection fraction of 60%. Moreover, right heart catheterization showed no signs of pulmonary hypertension. A repeat U/S and magnetic resonance imaging (MRI) of the abdomen showed no ascites or signs of liver cirrhosis. The MRI showed normal homogenous hepatic parenchyma with preserved architecture and a smooth non-nodular surface. Cirrhosis workup revealed a ferritin level of 50 ng/mL (30-160 ng/mL), ceruloplasmin level of 27 mg/dL (20-35), and normal serum copper level. A 24-hour urine collection for copper showed 23 µg (10-30). Serum alpha one antitrypsin (A1AT) level was within the normal range, and testing for anti-smooth muscle antibody (ASMA) and antimitochondrial antibodies (AMAs) was negative. Trans-vaginal U/S was done and showed no ovarian masses or suspicious findings of malignancy, thus excluding Meigs syndrome. Doppler U/S of the jugular and subclavian veins was done and ruled out venous thrombosis as a cause of transudative pleural effusion. Evaluation for multiple myeloma with the assessment of free light chains, urine, protein electrophoresis, and immunofixation was done but came back negative for paraproteinemia, making multiple myeloma less likely to be the culprit.

Furthermore, bilateral kidney U/S was done and displayed no signs of obstructive uropathy, and further investigation with a technetium 99m scan was negative for urinothorax. To exclude the possibility of amyloidosis, the patient underwent right-sided video-assisted thoracoscopic surgery (VATS) and was noted to have a 3.3×4 cm diaphragmatic defect. Cytology of the aspirated pleural fluid at that time was negative for malignant cells and pleural biopsy excluded amyloidosis and other pathological processes. Pleurodesis was not feasible owing to high output effusion. Finally, on duplex U/S of the abdomen, the umbilical vein was noted to be mildly tortuous, indicating recanalization caused by portal hypertension. However, there were no stigmata of thrombosis in the portal circulation. Put together, these findings indicated that portal hypertension was the most probable cause of the chylothorax.

Subsequently, the patient underwent trans-jugular liver biopsy with measurement of the portal pressures, which were significantly elevated at 18 mm Hg (5-10 mm Hg). This affirmatively confirmed the diagnosis of isolated hepatic hydrothorax. She underwent a trans-jugular intrahepatic portosystemic shunt (TIPS) procedure with a reduction of portosystemic pressure gradient from 18 mmHg to 6 mmHg. Post TIPS, the patient had a significant drop in the chylothorax output with major clinical improvement and resolution of her shortness of breath.

Liver biopsy exhibited early signs of cirrhosis with loss of the normal liver architecture, along with areas of bridging fibrosis and other areas demonstrating cirrhosis without significant steatosis. The portal tracts and fibrous bands showed chronic lymphocytic infiltrates with mild bile ductular reaction. The bile ducts were of normal architecture, and the periodic acid Schiff (PAS-D) stain was negative for cytoplasmic inclusions. The iron stain revealed no abnormal iron deposition.

A follow-up duplex abdominal U/S was done and disclosed a patent shunt with an appropriately directed flow in the hepatic vasculature. The chest tube was producing an output of 30-60 cc/24 hours, and she was able to tolerate chest tube clamping. Daily chest X-rays were stable with no evidence of substantial pneumothorax or pleural effusion, subsequently, the chest tube was removed. She was discharged home on furosemide and spironolactone without recurrence of her pleural effusion.

## Discussion

Chylothorax is a rare clinical entity that is characterized by the pathological accumulation of a triglyceride-laden fluid (chyle) within the pleural space. It is diagnosed by the identification of chylomicrons using lipoprotein electrophoresis of pleural fluid or a pleural fluid triglyceride level >110 mg/dL [2]. While chylothorax is an exudative effusion in the vast majority of cases, it should be distinguished from a transudative effusion. This distinction in the nature of the effusion is of diagnostic importance since only 1% of chylothorax cases are attributed to liver cirrhosis, often in association with the presence of chylous ascites [12].

Transudative chylothorax in the setting of decompensated cirrhosis is suggested to share a similar pathophysiology to that of ascites [13]. Edema, increased lymph flow, and collateral venous flow are compensatory physiological mechanisms responsible for portal hypertension dissipation at earlier stages before becoming clinically apparent at more advanced stages. This has been known as early as 1894, as Starling, investigating lymph flow mechanics, pointed out that venous obstruction does not result in sustained venous hypertension [14]. Dumont and Mulholland suggested that portal hypertension leads to an increase in pressures and lymph flow in the thoracic duct, which consequently causes extravasation of chyle into the pleural space [15]. The pressure gradient created by the negative intrathoracic pressure during inspiration favors the translocation of fluid from the peritoneal cavity into the pleural cavities. If the diaphragmatic defects are large enough and the rate of ascites formation does not exceed the rate of fluid translocation through the diaphragmatic defects, fluid will not collect in the peritoneal cavity.

**Figure 2:**
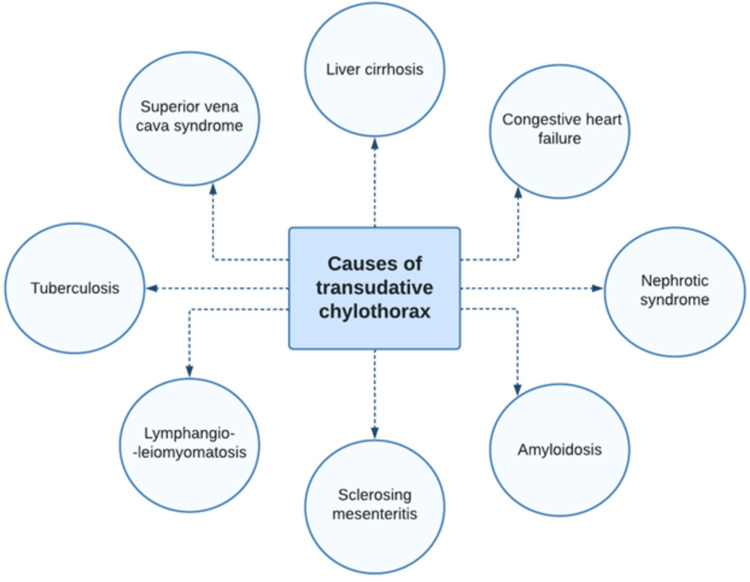
Etiologies of transudative chylothorax Information source: McGrath et al. (2010) [16]

Interestingly, transudative chylothorax has been reported in the literature in association with several identified conditions, of which liver cirrhosis was the most frequent (Figure 2). The majority of cases of chylothorax typically present with symptoms related to the underlying disease or concomitant ascites. While certain cases are discovered incidentally on chest imaging, symptomatic patients typically exhibit dyspnea and chest heaviness as a result of the mechanical impact of the effusion. Malnutrition and weight loss may arise in chronic cases owing to fat and protein loss, while prolonged depletion of immunoglobulins could lead to immune suppression [16]. On physical examination, dullness to percussion with reduced breath sounds over the affected area is commonly observed in patients with chylothorax. The initial step in investigating suspected chylothorax involves the confirmation of the diagnosis through fluid analysis. Traditionally, the fluid exhibits a milky coloration due to the high concentration of triglycerides in the form of chylomicrons. However, this feature can be misleading since only 50% of cases display the classic white milky appearance. The fluid can also present with serous, yellow, green, serosanguineous, or bloody appearances. The exact diagnosis of chylothorax is based on the identification of chylomicrons in the pleural fluid by lipoprotein electrophoresis. White blood cell differential of chylothorax reveals a predominance of lymphocytes. Pleural fluid demonstrates similar levels of glucose, protein, and electrolytes to that of plasma. Unless severe, there are no distinctive blood laboratory findings that are uniquely associated with chylothorax. Chest imaging reveals evidence of pleural effusion, which can occur unilaterally, either on the right side (52.3%), left side (25.7%), or bilaterally (22%), depending on the underlying etiology [17].

Cirrhotic chylothorax is initially managed conservatively. This includes dietary modification, repetitive thoracocentesis, diuretic use, and octreotide. A high-protein, low-fat diet decreases lymph formation [18]. TPN is an alternate option if diet modification fails. Octreotide, a somatostatin analog, decreases overall intestinal motility and absorption and has been found effective in diminishing the rate of chyle formation. If conservative management fails, pleurodesis can be used in cases of chylothorax with a low output. TIPS can be used as a last resort in treating refractory chylothorax due to cirrhosis. A systematic review published in 2015 reported that TIPS was effective in the management of 10 cases of chylothorax that were unresponsive to conservative management [19]. Although TIPS leads to rapid symptomatic improvement, the prognosis of patients with advanced liver disease after performing the procedure remains unchanged [20].

It is likely that our patient’s significant diaphragm defect had served as an outlet, ameliorating and delaying the systemic implications of portal hypertension, thus explaining why our patient presented with no stigmata of portal hypertension. This also explains the recalcitrant nature of this patient’s chylothorax, necessitating multiple drainage attempts and high chest tube output after placement, only to resolve following TIPS. This is further supported by two similar cases of cirrhosis-related chylothorax successfully treated by TIPS [21,22]. This affirms our belief that our patient’s chylothorax is portal pressure-dependent. Decreasing portal hypertension leads to a reduction in thoracic lymph flow and pressures, resulting in the cessation of chylothorax recurrence in our patient. TIPS, therefore, helps bring chylothorax recurrence to a halt in this patient population.

To date, only five cases presented with isolated chylothorax without coinciding ascites (Table 2). Two of them were previously diagnosed with alcoholic liver cirrhosis and portal hypertension [11,21,22]. This paper sheds light on occult portal hypertension as a potential cause of isolated transudative chylothorax whenever encountered. In a manner similar to cirrhotic ascites, transudative chylothorax results from hormonal and cytokine dysregulation leading to volume overload in the context of portal hypertension. Our research demonstrates that transudative chylothorax can sometimes replace ascites as the initial sign of liver cirrhosis and clinically significant portal hypertension. In such cases, chylothorax should be considered an ascites-equivalent in terms of both pathophysiology and treatment.

**Table 2:** Summary of all cases of isolated right-sided hepatic chylothorax in patients diagnosed with liver cirrhosis with no ascites TIPS: trans-jugular intrahepatic portosystemic shunt; TPN: total parenteral nutrition

Reported case	Age/Gender	Clinical presentation	Cirrhosis status	Ascites and stigmata of portal hypertension	Treatment	Outcome
Kirsch et al. [11]	38/m	Dyspnea, cough, scleral icterus, and pedal edema	Cirrhosis was diagnosed by percutaneous needle biopsy	Abdominal U/S confirmed the absence of ascites. He had no clinical signs of portal hypertension	Bed rest, 40 mg furosemide, and 100 mg spironolactone	Recovery
Kirsch et al. [11]	68/f	Dyspnea, non-icteric sclera, and splenomegaly	History of chronic alcoholic liver disease and cirrhosis	Abdominal U/S showed no ascites. Abdominal CT revealed splenic enlargement and diffuse abdominal varices	Bed rest and aggressive diuresis	Pleural effusion persisted despite aggressive attempts at diuresis
Kirsch et al. [11]	29/m	Dyspnea and bilateral lower extremity pitting edema	Abdominal CT showed features suggestive of liver cirrhosis	History of upper GI bleeding with +2 esophageal varices on endoscopy. Abdominal CT showed no ascites	The patient underwent a diagnostic and therapeutic right-sided thoracentesis only	The pleural effusion persisted and the patient died suddenly
Lutz et al, [21]	59/f	Dyspnea and cough	Alcoholic liver cirrhosis	Abdominal U/S showed splenomegaly but no ascites. Esophageal varices grade II were detected on upper GI endoscopy	Underwent diuretic therapy and TPN for 9 days; underwent TIPS for definitive therapy	Recovery
Kikoliski et al. [22]	84/f	Dyspnea	Cryptogenic liver cirrhosis	No ascites or clinical signs of portal hypertension	TIPS	Recovery

## Conclusions

Although hepatic chylothorax is not uncommon, very little is reported about isolated cirrhotic chylothorax, specifically when initial evidence of cirrhosis is lacking. After a broad evaluation, the diagnosis in this subset of patients often remains an enigma, prompting clinicians to look for objective signs of portal hypertension, despite the absence of suggestive clinical findings. In such cases, isolated transudative chylothorax with no ascites can rarely occur. This is because both transudative chylothorax and ascites in the setting of portal hypertension share similar pathophysiology related to fluid accumulation as a result of hormonal and cytokine dysregulation, as well as increased hydrostatic pressures caused by portal hypertension. Interestingly, the presence of a diaphragmatic defect plays a role in the development of isolated chylothorax in the context of cirrhosis. Finally, clinicians should suspect isolated portal hypertension and portal vein thrombosis in the absence of cirrhosis as a potential cause of transudative chylothorax to minimize diagnostic delays and initiate appropriate targeted therapies.

## References

[1] Sassoon CS, Light RW (1985). Chylothorax and pseudochylothorax. Clin Chest Med.

[2] Staats BA, Ellefson RD, Budahn LL, Dines DE, Prakash UB, Offord K (1980). The lipoprotein profile of chylous and nonchylous pleural effusions. Mayo Clin Proc.

[3] Doerr CH, Allen MS, Nichols FC 3rd, Ryu JH (2005). Etiology of chylothorax in 203 patients. Mayo Clin Proc.

[4] Apostolakis E, Akinosoglou K, Koletsis E, Dougenis D (2009). Traumatic chylothorax following blunt thoracic trauma: two conservatively treated cases. J Card Surg.

[5] Teng CL, Li KW, Yu JT, Hsu SL, Wang RC, Hwang WL (2012). Malignancy-associated chylothorax: a 20-year study of 18 patients from a single institution. Eur J Cancer Care (Engl).

[6] Romero S, Martín C, Hernandez L, Verdu J, Trigo C, Perez-Mateo M, Alemany L (1998). Chylothorax in cirrhosis of the liver: analysis of its frequency and clinical characteristics. Chest.

[7] Zamora-López MA, Farias-Navarro IC, Rendon-Ramirez EJ (2020). Chylothorax with transudate: an unusual presentation of tuberculosis. Eur J Case Rep Intern Med.

[8] Rice BL, Stoller JK, Heresi GA (2010). Transudative chylothorax associated with sclerosing mesenteritis. Respir Care.

[9] Koshima Y, Miyazaki S, Ninomiya J, Kuno Y, Ikeda T (2019). Transudative chylothorax associated with alcoholic cirrhosis. Oxf Med Case Reports.

[10] Karagiannidis A, Koulaouzidis A, Karavalaki M, Tan C (1991). Hepatic hydrothorax. Cause and management. Arch Intern Med.

[11] Kirsch CM, Chui DW, Yenokida GG, Jensen WA, Bascom PB (1991). Case report: hepatic hydrothorax without ascites. Am J Med Sci.

[12] Akbar A, Hendrickson T, Vangara A, Marlowe S, Hussain A, Ganti SS (2023). Hepatic chylothorax: an uncommon pleural effusion. J Investig Med High Impact Case Rep.

[13] Ginés P, Quintero E, Arroyo V (1987). Compensated cirrhosis: natural history and prognostic factors. Hepatology.

[14] Starling EH (1894). The influence of mechanical factors on lymph production. J Physiol.

[15] Dumont AE, Mulholland JH (1962). Alterations in thoracic duct lymph flow in hepatic cirrhosis: significance in portal hypertension. Ann Surg.

[16] McGrath EE, Blades Z, Anderson PB (2010). Chylothorax: aetiology, diagnosis and therapeutic options. Respir Med.

[17] Maldonado F, Hawkins FJ, Daniels CE, Doerr CH, Decker PA, Ryu JH (2009). Pleural fluid characteristics of chylothorax. Mayo Clin Proc.

[18] Takuwa T, Yoshida J, Ono S, Hishida T, Nishimura M, Aokage K, Nagai K (2013). Low-fat diet management strategy for chylothorax after pulmonary resection and lymph node dissection for primary lung cancer. J Thorac Cardiovasc Surg.

[19] Tsauo J, Shin JH, Han K, Yoon HK, Ko GY, Ko HK, Gwon DI (2016). Transjugular intrahepatic portosystemic shunt for the treatment of chylothorax and chylous ascites in cirrhosis: a case report and systematic review of the literature. J Vasc Interv Radiol.

[20] Garcia N Jr, Mihas AA (2004). Hepatic hydrothorax: pathophysiology, diagnosis, and management. J Clin Gastroenterol.

[21] Lutz P, Strunk H, Schild HH, Sauerbruch T (2013). Transjugular intrahepatic portosystemic shunt in refractory chylothorax due to liver cirrhosis. World J Gastroenterol.

[22] Kikolski SG, Aryafar H, Rose SC, Roberts AC, Kinney TB (2013). Transjugular intrahepatic portosystemic shunt for treatment of cirrhosis-related chylothorax and chylous ascites: single-institution retrospective experience. Cardiovasc Intervent Radiol.

